# Analysis by sex of safety and effectiveness of transvenous phrenic nerve stimulation

**DOI:** 10.1007/s11325-023-02882-5

**Published:** 2023-07-12

**Authors:** Soraya Samii, Scott McKane, Timothy E. Meyer, Neomi Shah

**Affiliations:** 1 Heart and Vascular Institute, Hershey Medical Center, Penn State University College of Medicine, Penn State Hershey HVI Mailcode H047, 500 University Drive, Hershey, PA 17033 USA; 2 ZOLL Respicardia, Inc., 12400 Whitewater Dr #150, Minnetonka, MN 55343 USA; 3 Division of Pulmonary, Critical Care and Sleep Medicine, Department of Medicine, Icahn School of Medicine at Mount Sinai, New York, NY USA

**Keywords:** Central sleep apnea, Transvenous phrenic nerve stimulation, Female

## Abstract

**Purpose:**

Little is known about sex differences in the treatment of central sleep apnea (CSA). Our post hoc analysis of the **rem**edē System Pivotal Trial aimed to determine sex-specific differences in the safety and effectiveness of treating moderate to severe CSA in adults with transvenous phrenic nerve stimulation (TPNS).

**Methods:**

Men and women enrolled in the **rem**edē System Pivotal Trial were included in this post hoc analysis of the effect of TPNS on polysomnographic measures, Epworth Sleepiness Scale, and patient global assessment for quality of life.

**Results:**

Women (*n* = 16) experienced improvement in CSA metrics that were comparable to the benefits experienced by men (*n* = 135), with central apneas being practically eliminated post TPNS. Women experienced improvement in sleep quality and architecture that was comparable to men post TPNS. While women had lower baseline apnea hypopnea index than men, their quality of life was worse at baseline. Additionally, women reported a 25-percentage point greater improvement in quality of life compared to men after 12 months of TPNS therapy. TPNS was found to be safe in women, with no related serious adverse events through 12 months post-implant, while men had a low rate of 10%.

**Conclusion:**

Although women had less prevalent and less severe CSA than men, they were more likely to report reduced quality of life. Transvenous phrenic nerve stimulation may be a safe and effective tool in the treatment of moderate to severe CSA in women. Larger studies of women with CSA are needed to confirm our findings.

**Clinical trial registration:**

ClinicalTrials.gov NCT01816776; March 22, 2013.

## Introduction

Central sleep apnea (CSA) is defined by cessations in breathing during sleep due to diminished or absence of ventilatory drive. CSA accounts for a small percentage of sleep apnea in the general population, with the majority of sleep apnea explained by obstructive respiratory events. The prevalence of CSA is tenfold higher in males compared to females [1], as demonstrated by data from the Sleep Heart Health Study. This was a prospective cohort study of sleep disordered breathing and risk of cardiovascular diseases that found only 0.2% of the females had CSA compared to 1.8% of the males [2]. However, CSA is most frequently found in individuals with heart failure, both with preserved ejection fraction (HFpEF) and with reduced ejection fraction (HFrEF). In a study of hospitalized patients with HFpEF, 29.5% were found to have CSA and only 13.7% of the patients with CSA were female [3]. In a study of hospitalized patients with HFrEF, 31% were found to have CSA and 18.3% of the patients with CSA were female [4]. Furthermore, evidence pertaining to the effect of treatment of CSA in females is lacking [1, 2, 5, 6]. Importantly, a recent study of patients with HF found that central apneas were associated with a worse prognosis in females than males [7].

Positive airway pressure (PAP) has shown inconsistent efficacy in treating CSA and has been most effective in patients with concomitant obstructive respiratory events. Adaptive servo-ventilation (ASV) was the standard non-invasive treatment for CSA. However, the randomized controlled “SERVE-HF” trial [8], which consisted of only 10% females, demonstrated that ASV increased the risk of cardiovascular death in patients with HFrEF. Further, the SERVE-HF trial showed that females had a non-significant increase in the relative risk of the primary endpoint (all-cause death or life-saving cardiovascular intervention plus unplanned hospitalization for worsening chronic heart failure) and cardiovascular death compared to control than males, although the incidence was higher for males.

Transvenous phrenic nerve stimulation (TPNS) is a non-PAP therapy that uses an implanted neurostimulator developed to treat moderate to severe CSA in adults. TPNS has been shown to restore a normal breathing pattern in patients by stimulating the phrenic nerve, resulting in consistent diaphragm contractions. The **rem**edē System Pivotal Trial was a randomized multi-center study that enrolled adult patients with moderate to severe CSA of any etiology except opioid-induced CSA. In this trial, the primary effectiveness endpoint was met with more TPNS active patients having a reduction in apnea hypopnea index (AHI) of ≥ 50% than the control group who had an implanted device that remained inactive for 6 months (51% TPNS active vs 11% control) [9]. At 12 months of therapy, 71% (5/7) of the females and 66% (31/47) of the males in the TPNS active group had a ≥ 50% reduction in AHI [9]. Yet, little has been published about sex differences in safety and other efficacy endpoints following the use of TPNS. Our post hoc analysis of the **rem**edē System Pivotal Trial aimed to present data on sex-specific differences in the safety and efficacy of TPNS for CSA.

## Methods

All patients enrolled in the **rem**edē System Pivotal Trial were included in this analysis. Details about the pivotal trial were previously published [10]. Briefly, patients underwent an overnight, in-laboratory, attended polysomnogram (PSG) at baseline and at 6-month intervals. The sleep studies were performed locally, and the study files were sent to a central core laboratory (Registered Sleepers, Winter Haven, FL) for scoring; enrollment decisions and effectiveness analyses used the core lab results. The Epworth Sleepiness Scale (ESS) questionnaire, a measure of daytime sleepiness with scores ranging from 0 to 24, with higher scores indicating more daytime sleepiness [11], was completed at baseline and 12 months. The patient global assessment (PGA) was a single question asked at 12 months: “Specifically in reference to your overall health, how do you feel today as compared to how you felt before having your device implanted: Markedly improved, moderately improved, mildly improved, no change, slightly worse, moderately worse, or markedly worse?”. The treatment group had TPNS therapy turned on 1 month after implant, and the control group had therapy activated following the randomized portion of the trial after 6 months. Control subject 6-month results prior to activation were used as a re-baseline for on therapy efficacy assessments. The data from both groups were pooled based on months of active therapy for this analysis in order to have a larger subset of patients on therapy for each sex. The effects of therapy are reported for males and females following 12 months of active therapy using efficacy endpoints from the pivotal trial [9], including the change in AHI, central apnea index (CAI), oxygen desaturation index (4%), and arousal index, percent of sleep in rapid eye movement (REM) and ESS, percentage of subjects with marked/moderate improved PGA, and percentage of subjects with a serious adverse event considered related to the implant procedure, device, or delivered therapy through 12 months.

In this exploratory analysis, nominal 2-sided *P*-values from Wilcoxon Signed-Rank tests for paired change from baseline within each sex are provided for continuous variables; however, due to the small sample size for females, the tests may be under-powered to detect statistically significant changes. Statistical tests comparing sexes were not performed due to lack of randomization and small sample size for females. Continuous endpoint results are presented as medians.

## Results

In the **rem**edē System Pivotal Trial, 11% (16/151) of the enrolled subjects were female, which represents the typical population of CSA in RCTs [7]. Of the sixteen enrolled female participants, 15 had therapy activated, as one did not successfully receive an implanted TPNS device and withdrew from the study. Five female participants did not have a PSG at 12 months of active therapy (1 death due to complication of cardiac surgery adjudicated as not related to the procedure, therapy, or device, 1 physician-initiated withdrawal due to poor health, 1 subject-initiated withdrawal due to mental health issues, 1 missed the visit due to poor health, and 1 declined the PSG). Four of these female subjects did not complete the questionnaires.

### Baseline demographics, comorbidities, and sleep characteristics

Baseline characteristics are displayed by sex in Table 1. Median age and BMI were 68 years and 28 kg/m^2^ for females compared to 65 years and 30 kg/m^2^ for males. Males had more comorbidities including hypertension (76% vs 69%), coronary artery disease (59% vs 38%), and heart failure (determined by baseline New York Heart Association Classification ≥ I) (64% vs 56%). A higher percentage of females than males had prior diagnosis of atrial fibrillation (50% vs 41%). Males had a higher AHI (median 44 vs 38 events/h) and CAI (median 24 vs 21 events/h) at baseline, but females had a higher ESS (median 12.5 vs 9.0).

**Table 1:** Baseline characteristics by sex

	Females (*N* = 16)	Males (*N* = 135)
Age (years)	68 (62, 75)	65 (59, 73)
Body mass index (kg/m^2^)	28 (26, 33)	30 (27, 35)
Heart rate (beats per minute)	76 (65, 81)	72 (64, 82)
Systolic blood pressure (mmHg)	126 (114, 131)	123 (111, 136)
Diastolic blood pressure (mmHg)	74 (69, 79)	73 (69, 84)
Hypertension	69%	76%
Coronary artery disease	38%	59%
Atrial fibrillation	50%	41%
Apnea hypopnea index ≥ 30 events/h (severe sleep apnea)	81%	81%
Apnea hypopnea index (events/hour)	38 (31, 57)	44 (32, 58)
Central apnea index (events/hour)	21 (14, 36)	24 (15, 41)
Oxygen desaturation index (events/hour)	34 (20, 47)	37 (25, 55)
Percent of sleep with oxygen saturation < 90% (%)	9.4 (1.9, 21.5)	9.1 (2.4, 18.8)
Arousal index (events/hour)	38 (24, 53)	43 (32, 59)
Epworth Sleepiness Scale (points)	12.5 (7.5, 14.5)	9.0 (5.0, 13.0)
Heart failure (New York Heart Class ≥ I)	56%	64%
NYHA class I/II/III/IV	25%/13%/19%/0	10%/29%/25%/0
Ejection fraction ≤ 45% (%)	47% (8/15)	61% (80/131)
Duration of implant (hours)	2.8 (2.2, 3.3)	2.6 (2.2, 3.3)
Stimulation lead side: left/right/not successful	44%/50%/6%	64%/33%/2%

Results presented as median (Q1, Q3) or percentage in category

### Polysomnographic efficacy measures

**Fig. 1:**
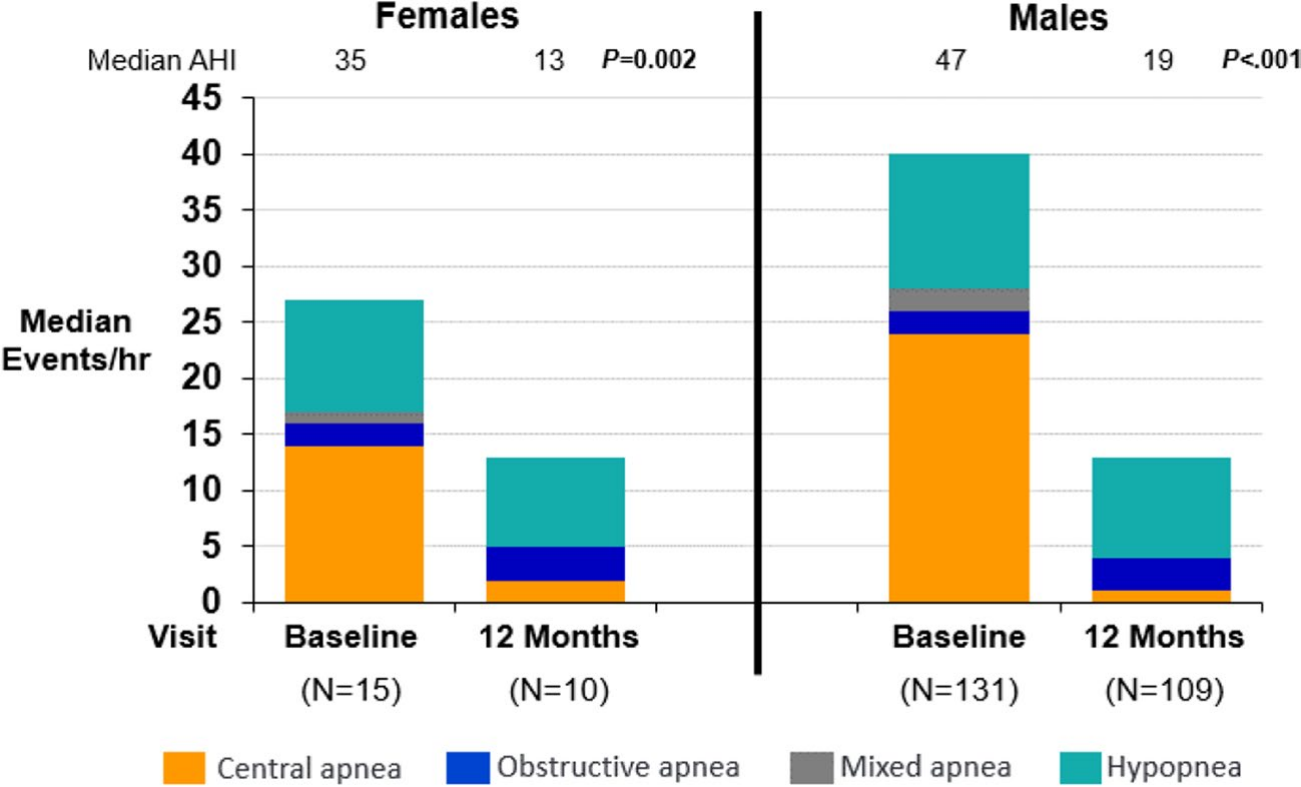
Changes in the apnea hypopnea index by sex. The apnea hypopnea index (AHI) components are shown at baseline and after 12 months of transvenous phrenic nerve stimulation. Females and males both had a large, statistically significant reduction in the AHI, with most of the improvement from the reduction in the central apnea index. The median (Q1, Q3) AHI paired change from baseline was − 21 events/h (− 24, − 10) in females (change from baseline *P* = 0.002) and − 22 (− 40, − 6) in males (*P* < 0.001), with corresponding changes in the central apnea index of − 14 (− 21, − 10) (*P* = 0.002) and − 21 (− 35, − 12) (*P* < 0.001). Abbreviations: AHI, apnea hypopnea index; hr, hour; *P*, *P*-value

After 12 months of active therapy, 60% (6/10) of females and 58% (63/109) of males achieved ≥ 50% reduction in AHI from baseline. The mean AHI was reduced at 12 months by 21 events/h for females and by 22 events/h for males. This change was mainly driven by a decrease in central apnea events to ≤ 2/h in each subgroup. Figure 1 displays the median components (central apneas, obstructive apneas, mixed apneas, and hypopneas) of the AHI at baseline and after 12 months of TPNS for females and males. The oxygen desaturation index (4%) improved by 20 events/h for females and 19 events/h for males. Table 2 presents effectiveness results.

**Table 2:** Effectiveness metrics after 12 months of therapy by sex

	Females			Males		
		12 months of therapy (*N* = 10)			12 months of therapy (*N* = 109)	
Endpoint^a^	Baseline (*N* = 15)	Result	Change from baseline	Baseline (*N* = 131)	Result	Change from baseline
Apnea hypopnea index (events/hour)	35 (23, 52)	13 (10, 23)	− 21 (− 24, − 10) 0.002	47 (33, 60)	19 (10, 34)	− 22 (− 40, − 6) < 0.001
Central apnea index (events/hour)	14 (11, 25)	2 (0, 5)	− 14 (− 21, − 10) 0.002	24 (14, 39)	1 (0, 4)	− 21 (− 35, − 12) < 0.001
Obstructive apnea index (events/hour)	2 (1, 3)	3 (1, 15)	1 (− 0, 5) 0.123	2 (1, 4)	3 (1, 7)	1 (− 1, 5) < 0.001
Mixed apnea index (events/hour)	1 (0, 2)	0 (0, 0)	− 0 (− 2, 0) 0.031	2 (0, 5)	0 (0, 1)	− 1 (− 4, − 0) < 0.001
Hypopnea index (events/hour)	10 (0, 23)	8 (5, 10)	− 3 (− 16, 6) 0.443	12 (4, 19)	9 (4, 19)	0 (− 6, 7) 0.860
Oxygen desaturation index 4% (events/hour)	31 (16, 57)	12 (10, 15)	− 20 (− 29, − 8) 0.004	40 (26, 58)	17 (8, 29)	− 19 (− 34, − 5) < 0.001
Percent of sleep in rapid eye movement (% of sleep)	6 (5, 16)	14 (8, 16)	4 (− 1, 7) 0.049	10 (6, 16)	15 (7, 22)	3 (− 3, 10) 0.001
Arousal index (events/hour)	38 (23, 40)	21 (17, 27)	− 12 (− 23, − 5) 0.014	41 (27, 60)	19 (14, 33)	− 15 (− 33, − 6) < 0.001
Percent of sleep with oxygen saturation < 90% (%)	7 (1, 34)	4 (1, 10)	− 5 (− 24, 0) 0.064	9 (3, 20)	4 (1, 14)	− 3 (− 9, 0) < 0.001
Percentage of subjects with marked/moderate improved patient global assessment	N/A	N/A	82% (9/11) (52%, 95%)	N/A	N/A	57% (64/113) (47%, 65%)
Epworth Sleepiness Scale (points)	12 (7, 14)	8 (5, 10) (*n* = 11)	− 2 (− 9, − 1) 0.008	9 (5, 14)	6 (3, 9) (*n* = 113)	− 3 (− 7, 0) < 0.001

*N/A* not applicable

^a^Median (Q1, Q3)/nominal 2-sided *P*-value from Wilcoxon Signed-Rank test for paired change from baseline to visit for continuous variables or percent (*n*/*N*) and 95% confidence interval for categorical variables

### Sleep quality

**Fig. 2:**
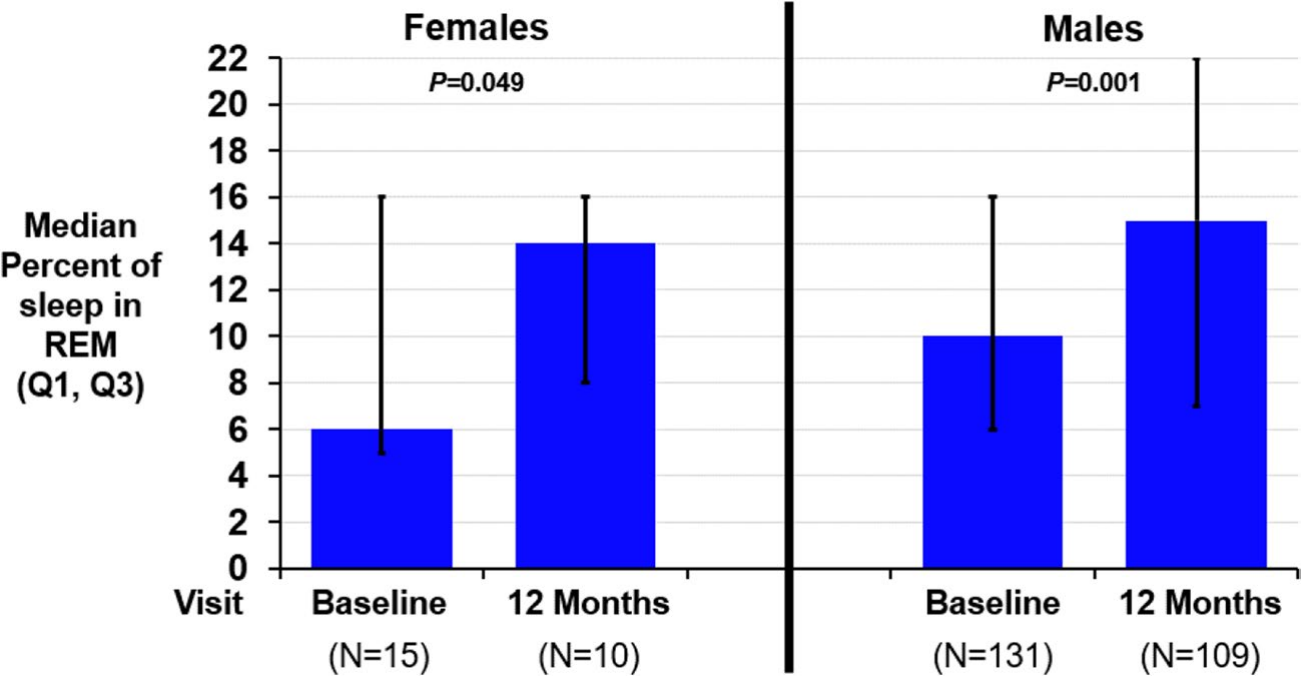
Changes in rapid eye movement sleep by sex. Rapid eye movement sleep increased for females and males after 12 months of transvenous phrenic nerve stimulation. The median (Q1, Q3) paired change from baseline was 4 (− 1, 7) for females (*P* = 0.049) and 3 (− 3, 10) for males (*P* = 0.001). Abbreviations: *P*, *P*-value; REM, rapid eye movement

The percent of REM sleep improved from baseline by a median of 3–4 percentage points in both females and males (Fig. 2 and Table 2). The arousal index improved similarly in both males and females (median 12–15 events/h). The percent of sleep with oxygen saturation less than 90% improved in males by 3 percentage points and in females by 5 percentage points.

### Quality of life assessment

**Fig. 3:**
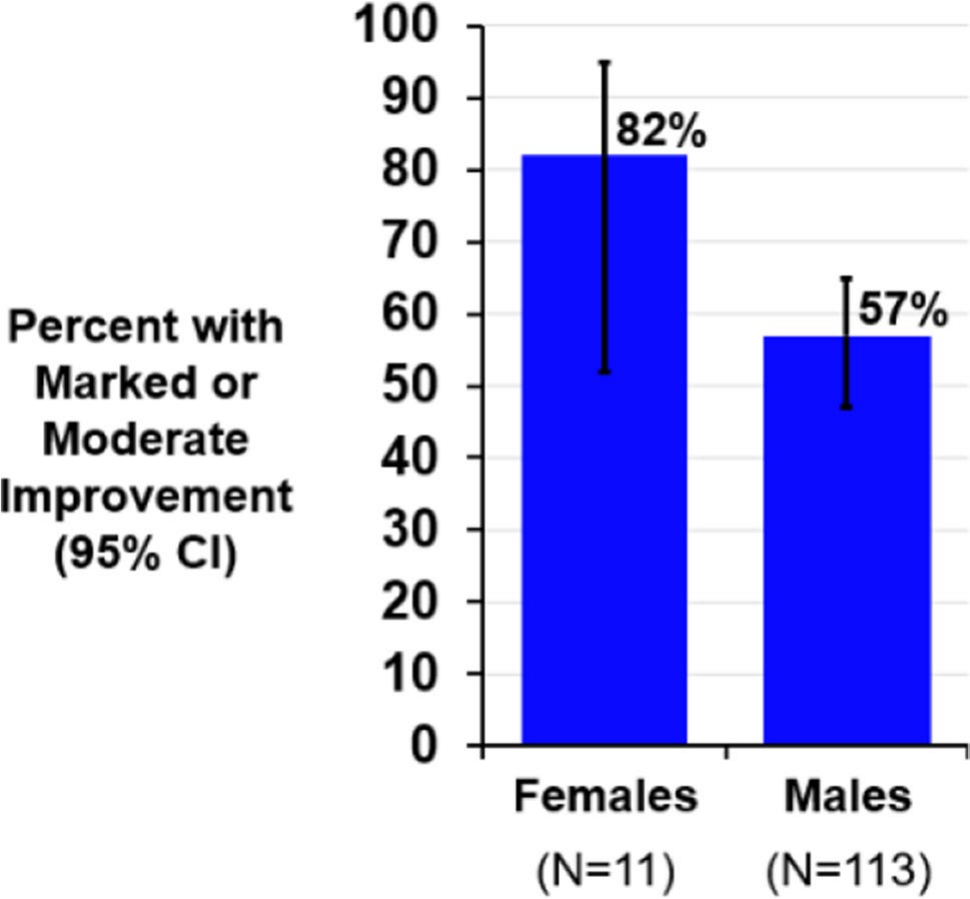
Patient global assessment by sex. The patient global assessment (PGA) is a single question asking “Specifically in reference to your overall health, how do you feel today as compared to how you felt before having your device implanted: Markedly improved, moderately improved, mildly improved, no change, slightly worse, moderately worse, or markedly worse?”. After 12 months of transvenous phrenic nerve stimulation therapy, 82% of females and 57% of males indicated marked or moderate improvement. Abbreviations: CI, confidence interval

**Fig. 4:**
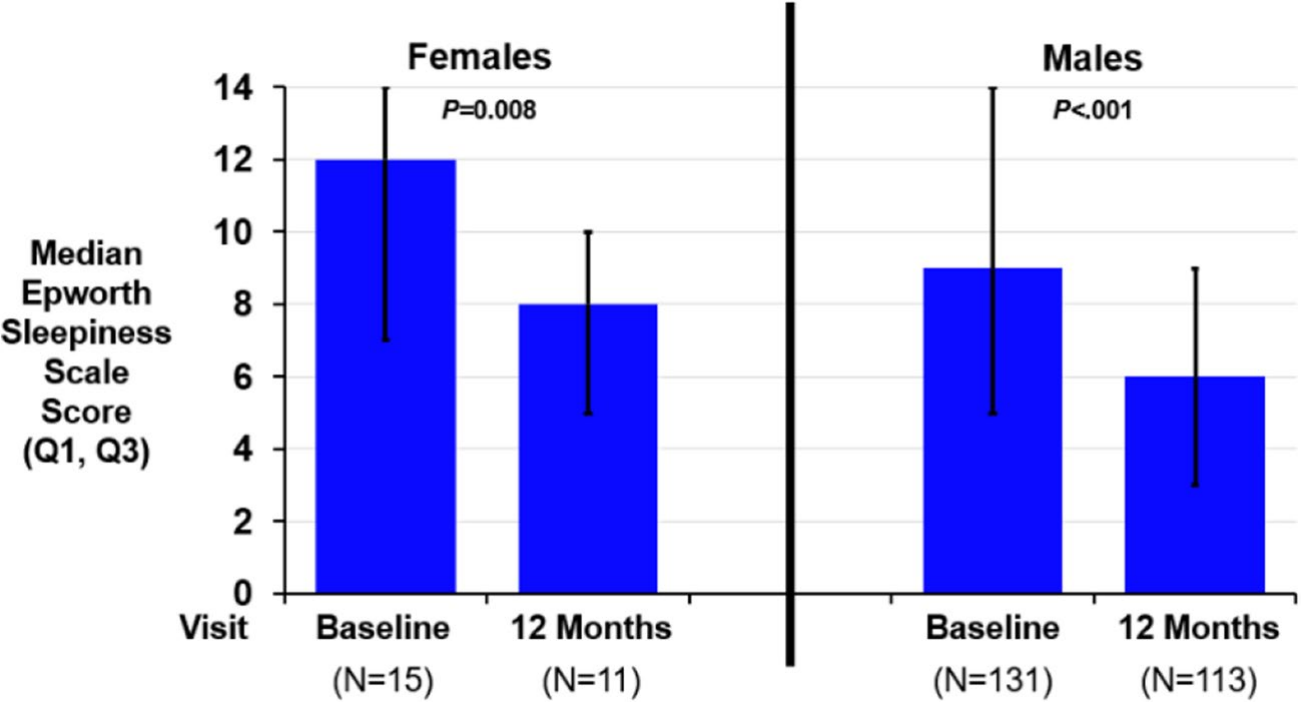
Changes in the Epworth Sleepiness Scale by sex. The Epworth Sleepiness Scale (ESS) is a measure of daytime sleepiness with scores ranging from 0 to 24. A score greater than 10 is considered excessive daytime sleepiness. The median (Q1, Q3) paired change from baseline was − 2 (− 9, − 1) for females (*P* = 0.008) and − 3 (− 7, 0) for males (*P* < 0.001) after 12 months of transvenous phrenic nerve stimulation. Abbreviations: *P*, *P*-value

Patient response was markedly or moderately improved on the PGA in 82% (9/11) of females and 57% (64/113) of males (Fig. 3 and Table 2). The ESS improved by a median of 2–3 points in males and females (Fig. 4 and Table 2).

### Cardiovascular

Left ventricular ejection fraction (LVEF) was measured at baseline and at 12 months of therapy. In this dataset, 11 females had completed assessments at baseline and at 12 months. There was an overall improvement in LVEF by 5% (3, 9). Males had an improvement of 2% (− 1, 6). Only four of the eight females with a heart failure diagnosis had data available at 12 months, so this subgroup analysis was not performed.

### Safety

At 12 months, 10% (13/135) of males and no (0/16) females experienced a serious adverse event considered related to the implant procedure, device, or delivered therapy. Implant procedural time in both groups was similar: median 2.8 h for females and 2.6 h for males (Table 1).

## Discussion

This is the first analysis specifically examining the outcomes of treating moderate to severe CSA with TPNS by sex. Our investigation found that females experience improvement in CSA from TPNS therapy, with central apneas being nearly eliminated, and this improvement is comparable to the benefit experienced by males. Additionally, females reported a 25-percentage point greater improvement in quality of life compared to males after 12 months of TPNS therapy. Also, females experienced improvement in sleep quality and architecture that was comparable to males. Finally, TPNS was found to be safe in females with no related serious adverse events reported through 12 months post-implant, while males had a low rate at 10%.

The **rem**edē System Pivotal Trial enrolled 16 females with moderate to severe CSA in the trial, accounting for 11% of the total subjects. Prior studies have suggested that females account for 9% of the population with CSA [1, 2] and 13–20% in females with heart failure [3, 4]. The number of females with moderate to severe CSA enrolled in this study, although low, was therefore expected and match those of other large clinical trials in CSA [7]. Baseline characteristics between females and males in our analysis were overall similar with some differences likely due to small sample size of the female group. It is worth noting that males had higher AHI and CAI than females, yet females had a higher ESS at baseline, suggesting a greater degree of sleepiness despite lower degree of CSA severity. Similar observations have been made comparing sexes with obstructive sleep apnea, where females have lower AHI but worse daytime sleepiness (higher ESS) [12, 13]. As previously reported, we found that both females and males with CSA benefit from TPNS with a reduction in AHI driven primarily by CAI reduction, which was similar in both sexes. Both sexes showed a similar improvement in the ESS score, and nearly all females (82%, 9/11) rated themselves as markedly or moderately improved on patient global assessment since baseline, whereas just over half (57%) of males declared that level of improvement. Sleep time spent with an oxygen saturation below 90% improved with TPNS in both sexes. This is an important secondary endpoint to follow in future studies as it is an independent predictor of all-cause mortality [14].

Our study has several limitations. First, the analysis was post hoc and was not powered to detect sex-specific differences in TPNS outcomes. The number of females in the analytical sample is low although it does seem to represent the prevalence of females with CSA in the population. Efforts to combine existing large datasets of CSA patients are needed to confirm the prevalence of CSA in females, and ongoing efforts to recruit females in clinical trials is critical. Some of the females in our cohort had missing data which further limits our ability to assess the endpoints. Because heart failure is a common condition associated with CSA, we wanted to explore the cardiovascular effects of TPNS on females. However, only four females with heart failure had 12-month follow-up data, limiting our analysis efforts. There continues to be a paucity of data on females with heart failure and CSA.

Strengths of our study include robust assessment and characterization of central sleep apnea using gold standard diagnostic polysomnography. Furthermore, we leverage a randomized clinical trial with pre-defined primary and secondary endpoints. A major strength of this study is its contribution towards understanding an under-investigated area in sleep medicine, namely, the impact of TPNS on qualitative and quantitative measures of sleep in females with CSA and the efficacy of non-PAP therapy for CSA in females. Based on our study findings, the prevalence of CSA in females is similar to other studies and diagnosed females are likely to be more symptomatic than males with increased daytime sleepiness and poor perceived quality of life. Our work provides novel insights on the clinical profile of females with CSA and the potential for improvement with TPNS.

In conclusion, although limited by small sample size, we demonstrate that TPNS may be an effective tool in the treatment of moderate to severe CSA in females. There is suggestion from our study that although females have less prevalent and less severe CSA than males, they are more likely to be symptomatic and report reduced quality of life compared to males. Females with CSA have greater degree of excessive daytime sleepiness, and therapy with TPNS markedly improves quality of life. Larger studies are needed to confirm our findings and to better characterize sex-specific differences in treatment response to PAP- and non-PAP-based therapies in females with CSA.

## Data Availability

The datasets generated during and/or analyzed during the current study are available from the corresponding author on reasonable request.
